# Upregulation of GLT-1 via PI3K/Akt Pathway Contributes to Neuroprotection Induced by Dexmedetomidine

**DOI:** 10.3389/fneur.2019.01041

**Published:** 2019-09-27

**Authors:** Mengyuan Peng, Xiaomin Ling, Ruixue Song, Xuan Gao, Zhifeng Liang, Fang Fang, Jing Cang

**Affiliations:** ^1^Department of Anesthesiology, Zhongshan Hospital, Fudan University, Shanghai, China; ^2^Comparative Nerve Imaging Study Group, Shanghai Institutes for Biological Sciences, Chinese Academy of Sciences, Shanghai, China

**Keywords:** dexmedetomidine, GLT-1, MCAO, MRI, PI3K/Akt, neuroprotection

## Abstract

Perioperative ischemic stroke usually leads to neurological dysfunction caused by neuron death. During the ischemic condition, excitotoxity due to extracellular glutamate accumulation is a main mechanism of neuron damage. The clearance of glutamate mainly depends on glutamate transporter-1 (GLT-1) which is expressed in astrocytes. Dexmedetomidine, an α2 adrenergic receptor agonist, is proved to induce neuroprotection. This study was set out to investigate the glutamate-related mechanism involved in the neuroprotective effect of dexmedetomidine. Middle cerebral artery occlusion (MCAO) was used as a model of ischemic stroke in our study. We determined Neurological deficit scores (NDS) and Magnetic resonance imaging (MRI) at three points (2, 6, and 24 h) after middle cerebral artery occlusion (MCAO) to evaluate the neuroprotective effect of dexmedetomidine. Besides, we performed western blot (6 and 24 h after MACO) and immunofluorescent staining (24 h after MCAO) to observe the expression of GLT-1. The effect and mechanism of dexmedetomidine on GLT-1 in primary cultured astrocytes were investigated using western blot and RT-PCR. Our results showed that pretreatment with dexmedetomidine improved NDS and reduced infarct volume as well as upregulating GLT-1 expression. Furthermore, using Atipamezole and LY294002, we found that dexmedetomidine significantly increased GLT-1 levels in astrocytes via activating α2 adrenergic receptor and PI3K/AKT pathway both *in vitro* and *in vivo* study. Overall, our present study indicated that dexmedetomidine had neuroprotective effects on ischemia stroke and upregulation of GLT-1 levels by PI3K/AKT dependent pathway might be the potential mechanism.

## Introduction

Perioperative stroke is defined as a brain infarction of ischemic or hemorrhagic etiology that occurs during surgery or within 30 days after surgery (1) and it's an important cause of morbidity and mortality perioperatively. Among all the perioperative stroke, ischemic stroke accounts for about 80% (2). Neurological and cardiovascular surgeries are associated with high risk of perioperative cerebral ischemia/reperfusion injury (3, 4). Especially in the cardiovascular surgeries, the overall incidence of stroke was estimated to be 2.0% to 10% (5). In non-cardiac and non-neurologic surgeries, perioperative stroke is also a potentially devastating complication with an incidence of 0.05–7% (6). Although rare, perioperative stroke is associated with an adjusted 8-fold increase in mortality (1). It also leads to functional impairments with 20% of survivors requiring institutional care and 15–30% being permanently disabled (7). Given its severe outcomes, in 2014, the Society for Neuroscience in Anesthesiology and Critical Care (SNACC) published a consensus statement on how to prevent perioperative stroke (8).

In the ischemic stroke, excitotoxity is a cardinal pathological mechanism which could result in neuron death and neurological dysfunction and it is mainly caused by extracellular glutamate accumulation in CNS (9, 10). The uptake of excessive extracellular glutamate relies on glutamate transporters. There are five glutamate transporter subtypes expressed in neurons or astrocytes. Among those transporters, glutamate transporters-1 (GLT-1), mainly expressed in the astrocytes, is the most important one responsible for up to 90% of glutamate clearance in brain tissue (11). Dysfunction or knockout of GLT-1 gene led to higher extracellular glutamate level, delayed neuronal death and remarkable increase in infarct areas in brain ischemic models (12). Therefore, GLT-1 may serve as a potential target for ischemic neurological deficits rescue.

Dexmedetomidine is an adrenoceptor alpha-2A agonist which is widely used as a sedation during perioperative period (13). It was demonstrated that the recovery of brain damage can be promoted by dexmedetomidine administration in animal models of traumatic brain injuries (TBI) and synaptic degeneration (14, 15). However, few investigations have thoroughly studied the neuroprotective effects of dexmedetomidine under ischemia condition and its potential mechanism.

So we hypothesized that the alpha-2A agonist dexmedetomidine would alter GLT-1 expression. The purpose of our study was to investigate the neuroprotective effects of dexmedetomidine, especially its effect on GLT-1 which is closely related to the cardinal pathological mechanism of ischemia stroke.

## Materials and Methods

### Drugs and Antibodies

P-Akt antibody (Cell Signaling, Cat#4060S, RRID: AB_2315049) and Akt antibody (Cell Signaling, Cat#4691S, RRID: AB_915783) were bought from Cell Signaling (Danvers, MA, USA). GLT-1 primary antibody (Abcam, Cat#ab41621; RRID: AB_941782) and β-actin (Abcam, Cat#ab16039, RRID: AB_956497) was purchased from Abcam (Cambridge, UK). Dexmedetomidine hydrochloride injection (ANDA: 209065) and ketamine (H32022820) was obtained from Hengrui Pharmaceutical Co. Ltd. (Jiangsu, China). The atipamezole (Selleck, Cat#S4650) and LY294002 (Selleck, Cat#S1105) was purchased from Selleck Chemicals (Houston, TX, USA). The Cytotoxicity Detection Kitplus (LDH) (Roche, Cat#No.04744926001) was bought from Roche. All the other reagents used were of analytical grade and procured from Sigma Chemicals unless mentioned otherwise.

### Animals

**Figure 1:**
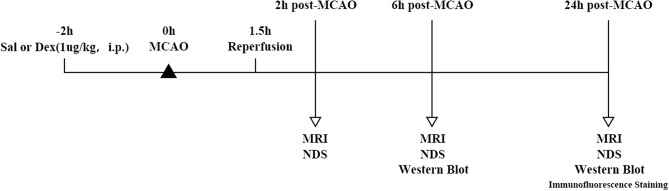
Timeline of MCAO model and experimental parameter. SD rats were divided into four groups randomly: Sal + Sham, Sal + MCAO, Dex + Sham, Dex + MCAO. After 2 h pretreatment of Normal saline (1 ml/kg, i.p.)/Dex(1 ug/Kg, i.p.), Sham surgery/MCAO model was performed. Reperfusion took place after 1.5 h of MCAO. Rats underwent 3 times MRI analyses at 3 time points and neurological deficit score (*n* = 10 animals per group, but 2 rats were excluded because missed detection time. So, the sample size included in the statistical analysis is *n* = 8) at 2 h post-MCAO, 6 h post-MCAO, and 24 h post-MCAO. Western blotting analysis of GLT-1 expression was performed at 6 h (*n* = 10 animals per group) and 24 h (*n* = 10 animals per group) post-MCAO and the expression of GLT-1 was observed by immunofluorescence staining at 24 h (*n* = 3 animals per group) post-MCAO.

Male Sprague-Dawley rats weighing 200–240 g were procured from the Experimental Animal Center of Fudan University and group housed in polypropulene cages with 4 animals per cage. The animals were maintained under standard laboratory conditions with natural dark-light cycle (14 ± 1 h light; 10 ± 1 h dark). They were feed by standard dry rat diet (Aashirwad industries, Chandigarh) and pure water. The study was not pre-registered. To minimize the animal suffering, we replaced the wood filings in the cage every day during the experimental procedures. Animals were deeply anesthetized with sodium pentobarbital (40 mg/kg, i.p) before surgical procedures. Sample sizes were performed using a calculation program available from http://powerandsamplesize.com/Calculators/, with 95% confidence interval and 80% power for studies using effect sizes observed in previous study (16). SD rats were divided into 4 groups randomly (*n* = 10 animals per group): Sal + Sham, Sal + MCAO, Dex + Sham, Dex + MCAO. After 2 h pretreatment of Normal saline (1 ml/kg, i.p.)/Dex(1 ug/Kg, i.p.), Sham surgery/MCAO model was performed. For the studies of the immunofluorescent staining, the number of rats was *n* = 3. The experimental design of MCAO model is described as Figure 1. Inclusion criteria included male Sprague Dawley rats with weight 200 ± 240 g. Exclusion criteria include behavioral defect or diseased animals and animals that was not measured in time. The animal protocol was approved by the Ethics Review Committee for Animal Experimentation of Fudan University (Shanghai, China) and the approve number was 20170223-098.

### MCAO Model and Reperfusion Injury

We established middle cerebral artery occlusion (MCAO) model to induce the cerebral ischemic state. The middle cerebral artery was occluded for 90 min using an intraluminal filament technique as described previously under pentobarbital sodium anesthesia (40 mg/kg, i.p.) (17). A midline incision was made and the left common carotid artery, external carotid artery and internal carotid arteries were exposed. The diameter of the monofilament nylon was 0.24 mm and its tip rounded by silica gel. It was used to occlude the MCA. The filament was inserted from the left external carotid artery into the lumen of the internal carotid artery up to 20 mm until a resistance was felt which meant the occlusion at the origin of MCA. The nylon filament was allowed to remain in the place for 90 min and then gently retracted to reperfusion. In sham operated rats, the filament was put into the external carotid artery but was not inserted into the internal carotid artery (17). The regional cerebral blood flow (rCBF) was monitored by laser Doppler flowmetry (PeriFlux 5000; Perimed AB, Sweden). If the rCBF showed a sharp drop below 30% and recovered up to more than 80% of baseline level, the MCAO model was considered effective (18). During the whole process of experiment, the temporal temperature was maintained at 37 ± 1°C by a thermostatic blanket.

### Neurological Deficit Score (NDS)

To assess brain function status after MCAO, we performed behavioral monitoring and NDS scores. NDS of rats was evaluated by an investigator blinded to the treatment on three time point (2 h post-MCAO, 6 h post-MCAO, and 24 h post-MCAO) using a standard 6-point scale as: 0 = no neurological deficits, 1 = failure to extend the right forepaw fully, 2 = circling to the right, 3 = paresis to the left, 4 = no spontaneous walking and 5 = death (19).

### Magnetic Resonance Imaging (MRI) and Assessment of Infarct Volume

MRI studies were carried out using an animal MRI 9.4T scanner (Bruker, BioSpec 94/30 US/R). The MRI instrument was equipped with 72 mm transmit only ^1^H circularly polarized coil with transmit only capability (112/72 mm, outer/inner diameter) in combination with rat brain surface coil, ^1^H circularly polarized coil with receive only capabilities. MRI (DWI and T2WI) started at 0.5 h after reperfusion (2 h post-MCAO) and was continued 6 and 24 h post-MCAO. In each animal DWI was acquired using a spin-echo echo-plannar-imaging sequence with the following parameters: silce thickness (THK) = 2 mm, number of silces (NS) = 5, field of view (FOV) = 32 × 32 mm, matrix = 128 × 128, repetition time (T_R_) = 2,000 ms, echo time (T_E_) = 18.453 ms, number of averages (NA) = 2, b values = 400, 600, 800 and 1,000 s/mm^2^, δ = 5 ms, Δ = 15 ms, G = 118 mT/m. T2WI was acquired using the multi-spin multi-echo sequence (FOV = 32 × 32 mm, matrix = 256 × 256, THK = 2 mm, NS = 5, NA = 1, number of echoes = 12, T_R_ = 2,500 ms, T_E_ = 33 ms). Image data were transferred to a software called RadiAnt DICOM viewer. From T2WI MRI, we calculated volume of infarction and the lesions in T2WI was defined as hyperintense areas on the image with the highest T2 weighting (TE = 96 ms) (20).

Infarct volume was assessed by an investigator blinded of the experimental grouping using Image J software. The Infarct volume was calculated as (S1 + S2 + S3 + …… + S18) × 1 mm^3^, where S_1_ to S_18_ represented slice infarct area in mm^2^ each with a slice thickness of 1 mm. If there was no infarction on the slice, Sn = 0. Corrected infarct volume was calculated by formula = (infarct volume × contralateral volume)/ipsilateral volume (18, 21).

### Immunofluorescent Histochemistry

To directly observe the effect of dexmedetomidine on GLT-1 expression, we performed immunofluorescence staining on brain slices. At designated time points after drugs' administration, animals were anesthetized and perfused through the ascending aorta with 200 ml of 0.9% saline solution, followed by 400 ml of 4% paraformaldehyde (PFA) in phosphate buffer (PB, pH 7.4). After perfusion, the brains were removed and post-fixed in the same fixative for 2–4 h, then dehydrated for 24 h at 4°C in 0.1 M PB containing 20% sucrose and another 24 h in 0.1 M PB containing 30% sucrose. Coronal sections (25 um) were continuously cut at the level of the dorsal hippocampus (3.3–4.0 mm posterior from bregma) with a cryotome, collected in 0.01 M PBS (PH 7.4) and then processed for immunofluorescent staining. Sections were blocked with 10% goat serum in 0.01 M PBs containing 0.3% Triton X-100 for 2 h at room temperature, and then incubated overnight at 4°C with the primary antibodies: mouse anti-Glial Fibrillary Acidic Protein (GFAP)(1:200, Abcam, Cambridge, UK) mixed with rabbit anti-GLT-1(1:200, Abcam, Cambridge, UK). After washed three times, the sections were then incubated for 2 h at room temperature with Alexa Fluor® 594-conjugated goat anti-mouse/rabbit IgG (1:500, Abcam, Cambridge, UK). Nuclei were stained with 0.01 M PBS containing 10 ug/ml 4′,6-diamidino-2-phenylindole (DAPI) after the last wash. Images were obtained using a fluorescence microscope (FV1000; Olympus, Tokyo, Japan) and the observation area was the hippocampus. Since the background is high in the image, the quantification of GLT-1 immunohistochemistry data was difficult. Images of immunofluorescence staining were showed, but no quantitative calculation was performed.

### Primary Astrocytes Culture

GLT-1 mainly expressed in the cerebral astrocytes. To clarify the effect and mechanism of dexmedetomidine on GLT-1, we performed primary astrocytes culture. Primary astrocytes were obtained from the cortex and hippocampus of newborn (<24 h) Sprague-Dawley rats, according to the procedures described previously with some modifications (22). Briefly, rats were sacrificed by decapitation, and each brain was carefully removed to an ice-cold Hank's magnesium and calcium-free solution. After trypsinization, the dissociated cells were passed through sterile nylon meshes and then resuspended in Dulbecco's-modified eagle medium (DMEM, GIBCO, Cat#41965062) supplemented with 10% heat-inactivated fetal bovine serum (FBS, GIBCO, Cat#16140071). Then the cells were seeded to 6-well plates at a density of 1 × 10^6^ cells and cultured for 10–14 days at 37°C in a humidified incubator (5% CO_2_/95% air). The medium was renewed every 3 days. Microglia and oligodendrocytes were removed by shaking at 260 pm overnight at 37°C in an orbital shaker. Cells in the culture were shown to be astrocytes with a purity of 97 ± 2% after characterization by immunostaining using a primary anti-GFAP antibody.

### Protein Extraction and Western Blotting

To quantify the GLT-1 expression, western blotting was performed on primary hippocampi of rat brain and astrocytes, respectively. According to the western blotting protocol previously published (23), the astrocytes/hippocampi were harvested on ice in the RIPA lysis buffer (50 mM Tris, 150 mM NaCl, 0.1% SDS, 1% Triton X-100, 1% sodium deoxycholate, sodium orthovanadate, sodium fluoride, EDTA, leupeptin, pH 7.4) (Cat#P0013B, Beyotime Biotech Inc, Shanghai, China) containing 0.5 mM phenyl methyl sulfonyl fluoride (PMSF) after the drug's treatments. Samples were stored at −80°C for Western blot analysis. The protein concentration of each protein extract sample was determined by the BCA method, and the sample volume was calculated according to the concentration to ensure the consistency of the total protein amount (40 ug) of each sample. Then, protein material was electrophoresed on a 10% SDS-PAGE and subsequently transferred to nitrocellulose filter (NC) membrane (Millipore, Billerica, MA, USA). The membrane was put in blocking buffer (LI-COR, USA) at room temperature for at least 1 h and then incubated with the primary antibody (GLT-1 at 1:800; p-AKT at 1:1000; AKT at 1:1,000 and β-actin at 1:1000) in primary antibody dilution buffer (Cat#P0023A, Beyotime Biotech Inc, Shanghai, China) at 4°C overnight. The membrane was washed three times with TBST [tris-buffered saline (TBS) and 0.1% Tween 20] and then incubated with horseradish peroxidase (HRP)-conjugated secondary antibody at room temperature for another 1 h. Wash 3 times again. Specific signals of proteins were visualized by Near Infrared Two-color Laser Imaging System (LI-COR, USA). The bands obtained on the films were quantified by densitometry (Image J 5.0) to determine the expression of the protein. Beta-actin was taken as loading control for all the immunoblotting experiments, and the ratio was calculated.

### Reverse Transcription-Polymerase Chain Reaction (RT-PCR)

Astrocytes was collected after each drug's treatment. RT-PCR was performed as described (24). Total RNA of the astrocytes was extracted using Cell Lysis Buffer (EZBioscience, USA) according to the manufacturer's instructions. 2 ug RNA was taken for reverse transcription using EZ-press Cell to Ct Kit (EZBioscience, USA). The PCR amplification was carried out as follows: a 42 min incubation at 42°C for cDNA synthesis, a 5 min hot-start at 95°C followed by 40 cycles of denaturation at 95°C for 10 s, annealing and extension at 60°C for 40 s. The total volume of PCR system was 20 ul. Data were normalized to β-actin expression. Forward/reverse primers were: GLT-1 5′-CAAGCTGATGGTGGAGTTCTT-3′/5′-CACGCTTGTCAATCCCTAGAT-3′; β-actin 5′-TGTGGCATCCATGAAACTACA-3′/5′-CCACCAATCCACACAGAGTAC-3′.

### Statistical Analysis

Graph pad prism 5.0 version (GraphPad Software Inc., San Diego, CA, USA) was used for statistical analyses. Statistical analysis was performed by *t*-test and two-way ANOVA analysis followed by the Bonferroni multiple comparisons test. *P* < 0.05 represents statistically significant. Data are expressed as the mean ± SD.

## Results

### Dexmedetomidine Pre-treatment Attenuated Cerebral Ischemic Injuries

**Figure 2:**
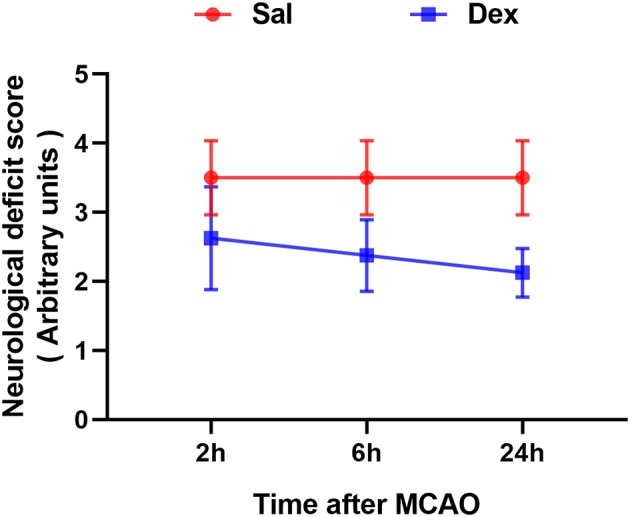
Dexmedetomidine pretreatment reduced the neurological deficit score (NDS) of rats experienced MCAO. Neurological deficit score assessed after MCAO. Dexmedetomidine pretreatment significantly attenuated the NDS after MCAO, but it didn't in a time-dependent manner. Data are expressed as mean ± SD (*n* sample = 8 animals per group).

**Figure 3:**
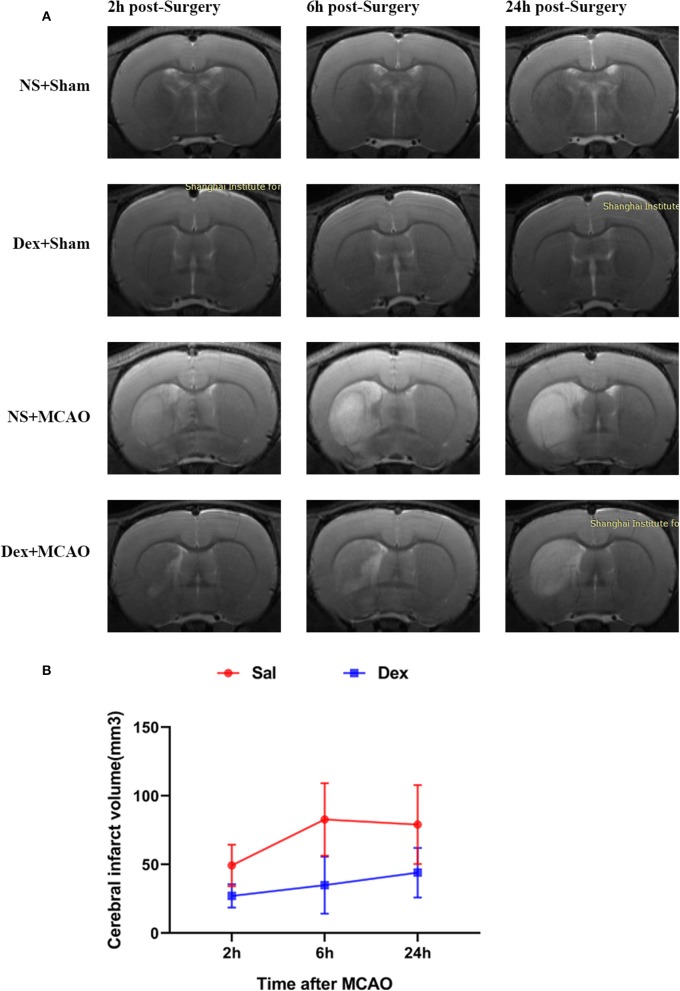
Dexmedetomidine pretreatment reduced the cerebral infarct volume. MRI analysis was conducted at 2, 6, and 24 h after MCAO. **(A)** T2-weighted MRI images of brain from 4 groups at 2, 6, and 24 h post-MCAO. **(B)** Dexmedetomidine pretreatment significantly reduced the infarct volume after MCAO, but it didn't in a time-dependent manner. Infarct volume are expressed as mean ± SD (*n* sample= 8 animals per group).

All rats survived after surgery and met the inclusion criteria (the rCBF showed a sharp drop below 30% and recovered up to more than 80% of baseline level) of MCAO model. To assess brain function status after MCAO, behavioral monitoring and NDS scores were performed at 2, 6, and 24 h after MCAO. Scores of Sal + Sham and Dex + Sham groups were 0 and Sal + MCAO groups were same with each other at 3 time points as shown in Figure 2, which indicated that neither dexmedetomidine nor the surgery itself caused time-dependent changes in neurological deficit scores (NDS). Dexmedetomidine pretreatment significantly attenuated the NDS after MCAO, but it didn't in a time-dependent manner (drug factor: *F*_1,42_ = 50.52, *p* < *0.0001*; time factor: *F*_2,42_ = 0.8317, *p* = *0.4424*; interaction: *F*_2,42_ = 0.8317, *p* = *0.4424*). To assess the infarct volume of brain, MRI were taken after surgery (Sham/MCAO) pretreated with drug (Sal/Dex). In Sal/Dex+Sham groups at 3 time points, no infarction was found. As shown in Figure 3, in Sal/Dex+MCAO groups, time was considered affect the infarct volume. And dexmedetomidine pretreatment significantly attenuated the infarct volume after MCAO, but it didn't in a time-dependent manner—the interaction between drug and time was considered not significant (drug factor: *F*_1,42_ = 34.25, *p* < *0.0001*; time factor: *F*_2,42_ = 6.080, *p* = *0.0048*; interaction: *F*_2,42_ = 1.522, *p* = *0.2301*).

### GLT-1 Was Upregulated by Dexmedetomidine in MCAO Model

**Figure 4:**
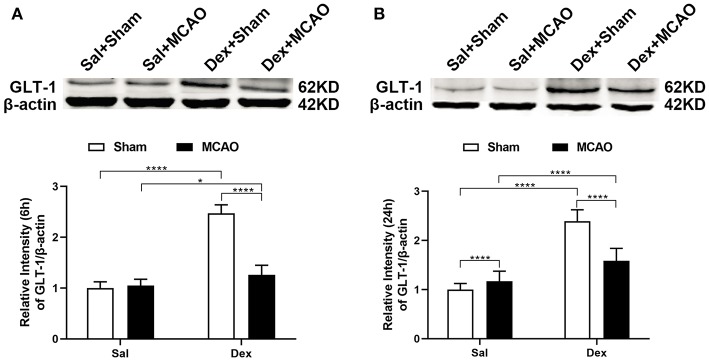
Dexmedetomidine upregulated GLT-1 expression in rat brains. Western blotting analysis of GLT-1 expression at 6 h **(A)** and 24 h **(B)** following rat MCAO. Dexmedetomidine significantly upregulated GLT-1 expression in Sham group at 6 h (*t* = 23.38, *p* < *0.0001*) and 24 h (*t* = 16.35, *p* < *0.0001*), and in MCAO group at 6 h (*t* = 3.338, *p* = *0.0103*), and 24 h (*t* = 4.921, *p* < *0.0001*) in rat brains. Data are expressed as mean ± SD (*n* = 10 animals per group). *p < *0.05*, ****p < *0.0001* (two-way ANOVA followed by the Bonferroni multiple comparisons test).

**Figure 5:**
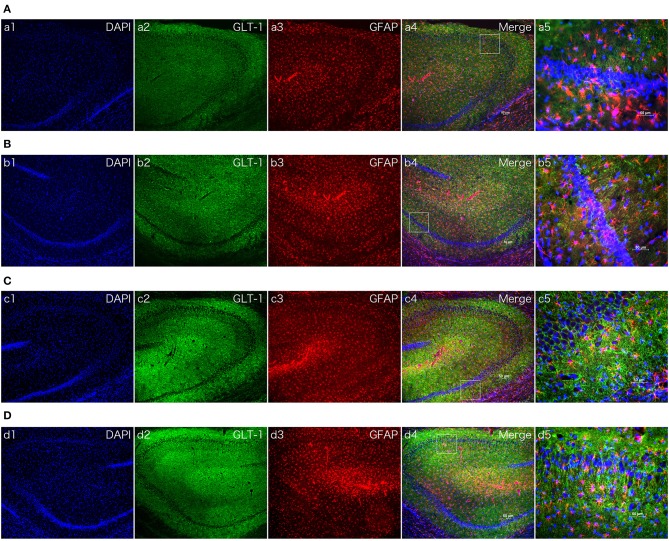
The effect of dexmedetomidine in hippocampi of MCAO model rats (24 h). Images of immunofluorescent staining: GFAP (red), GLT-1 (green), and DAPI (blue). GLT-1 signal in Dex + Sham and Dex + MCAO was more intensive than Sal groups. **(A)** Saline + Sham group. **(B)** Saline + MCAO group. **(C)** Dex (1 ug/Kg) + Sham group. **(D)** Dex (1 ug/Kg) + MCAO group. a,b,c,d 1~4 were 10×, a,b,c,d 5 was 40×. Scale bars = 50 um.

To characterize the GLT-1 levels after MCAO, brain tissue samples were collected from MCAO and Sham-operated animals at 6 and 24 h after MCAO. In Figure 4, Western Blotting revealed that dexmedetomidine significantly upregulated GLT-1 expression at 6 h (injury factor: *F*_1,44_ = 171.0, *p* < *0.0001*; drug factor: *F*_1,44_ = 356.9, *p* < *0.0001*; interaction: *F*_1,44_ = 200.8, *p* < *0.0001*) and 24 h (injury factor: *F*_1,44_ = 28.12, *p* < *0.0001*; drug factor: *F*_1,44_ = 226.2, *p* < *0.0001*; interaction: *F*_1,44_ = 65.32, *p* < *0.0001*) in rat brains. In addition, GLT-1 in Dex + MCAO groups were lower than Dex + Sham groups at the two time points (*p* < *0.0001*). It indicates that GLT-1 was decreased after MCAO, which is consist with previous reports (25). To confirm the effect of dexmedetomidine on GLT-1, we conducted immunofluorescent staining. From the images in Figure 5, we found that the GLT-1 signal in Dex + Sham and Dex + MCAO was intensive. The results indicated that dexmedetomidine could increase the expression of GLT-1, which were consist with the western blot above.

### Dexmedetomidine Upregulated GLT-1 Expression and the Levels of Phospho-Akt/Akt in Primary Astrocytes

**Figure 6:**
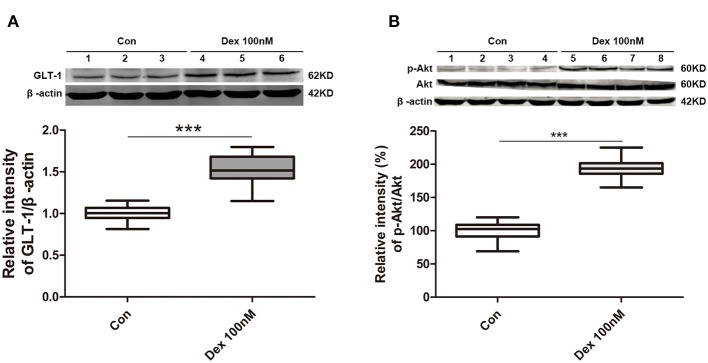
Dexmedetomidine upregulated GLT-1 expression and the levels of phospho-Akt/Akt in primary astrocytes. **(A)** Dexmedetomidine significantly upregulated GLT-1 at 100 nM for 30 min (*p* < *0.001*). **(B)** Phospho-Akt was obviously increased after of dexmedetomidine treatment and the ratio of p-Akt/Akt in Dex group was increased (*p* < *0.001*). Box plots show the median, interquartile, and range, considering control group mean values as 100% (*n* sample = 12 cell wells per group, 3 independent cell preparations were performed). ***p < *0.001* vs. Control group.

We next investigated whether dexmedetomidine can activate GLT-1 and its mechanisms. We intended to explore the underlying mechanisms with cell experiments. GLT-1 mainly expressed in astrocyte, so we chose astrocytes as our object cells. From the results in Figure 6A, we found dexmedetomidine significantly upregulated GLT-1 at 100 nM for 30 min (*p* < *0.001*). The action time and concentration of dexmedetomidine were determined from the preliminary experiment.

PI3K/Akt pathway is important and mediates some crucial regulation of cell survival. Especially, PI3K/Akt pathway is known to induce GLT-1 expression (16). Therefore, we observed whether dexmedetomidine-induced GLT-1 upregulation was mediated through PI3K/Akt pathway in cultured primary astrocytes. Western Blotting showed that the level of phospho-Akt was obviously enhanced after the treatment of dexmedetomidine and the ratio between p-Akt and Akt in Dex group was increased (*p* < *0.001*) (Figure 6B). These results suggested that dexmedetomidine can activate PI3K/Akt pathway.

### Atipamezole and LY294002 Reversed the Upregulation of GLT-1 Induced by Dexmedetomidine

**Figure 7:**
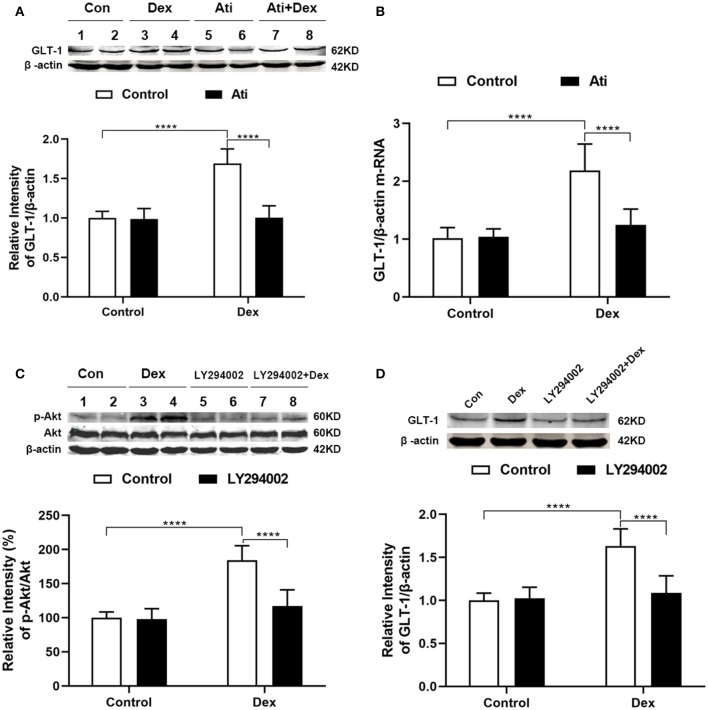
Atipamezole and LY294002 reversed the upregulation of GLT-1 induced by dexmedetomidine. Cultured astrocytes were harvested after drugs administration (Dex, 100 nM, 30 min; Atipamezole, 300 nM, 1 h). **(A,B)** Western blot and RT-PCR showed that the upregulation of GLT-1 induced by dexmedetomidine dramatically reversed by Atipamezole (*t* = 11.84, *p* < *0.0001*) (*t* = 7.965, *p* < *0.0001*), respectively, while Atipamezole did not affect the GLT-1 protein and mRNA itself. **(C,D)** Western blotting showed that LY294002 reversed the upregulation of p-Akt/Akt (*t* = 9.088, *p* < *0.0001*) and GLT-1 (*t* = 8.335, *p* < *0.0001*) induced by dexmedetomidine. Data are expressed as mean ± SD (*n* sample = 12 cell wells per group, 3 independent cell preparations were performed). ****p < *0.0001* (two-way ANOVA followed by the Bonferroni multiple comparisons test).

In order to analyze the detailed mechanism of between dexmedetomidine and GLT-1, we chose Atipamezole and LY294002 as tools. Atipamezole is an α2 adrenergic receptor antagonist. Results from western blot showed that the upregulation of GLT-1 induced by dexmedetomidine dramatically reversed by atipamezole (Atipamezole factor: *F*_1,44_ = 72.82, *p* < *0.0001*; Dexmedetomidine factor: *F*_1,44_ = 74.25, *p* < *0.0001*; interaction: *F*_1,44_ = 67.42, *p* < *0.0001*) (Figure 7A). RT-PCR was consistent with western blot results (Figure 7B).

LY294002, an inhibitor of PI3K pathway, was used to confirm that PI3K/Akt pathway was involved in the mechanism. We measured the levels of p-Akt, Akt, and GLT-1 protein by Western Blotting (Figures 7C,D). LY294002 did not affect the p-Akt and GLT-1 protein itself (*p* > 0.05). Dexmedetomidine significantly upregulated the p-Akt/Akt ratio, but the upregulation of p-Akt/Akt induced by dexmedetomidine was dramatically reversed by LY294002 (LY294002 factor: *F*_1,44_ = 43.78, *p* < *0.0001*; Dexmedetomidine factor: *F*_1,44_ = 96.79, *p* < *0.0001*; interaction: *F*_1,44_ = 38.89, *p* < *0.0001*). GLT-1 level shows the similar trend as the ratio of p-Akt/Akt in each group (LY294002 factor: *F*_1,44_ = 31.87, *p* < *0.0001*; Dexmedetomidine factor: *F*_1,44_ = 56.15, *p* < *0.0001*; interaction: *F*_1,44_ = 37.73, *p* < *0.0001*). The results indicated that LY294002 could reduce the upregulation effect of dexmedetomidine on GLT-1 by inhibiting the PI3K/Akt pathway.

### Dexmedetomidine Upregulated GLT-1 Expression in Hippocampi of Normal SD Rats

**Figure 8:**
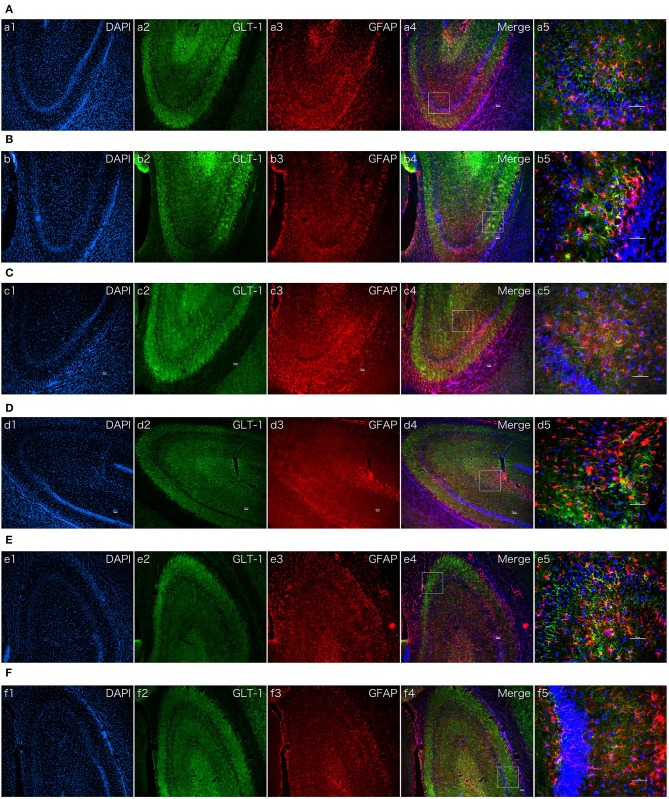
The effect of dexmedetomidine, Atipamezole, and LY294002 in hippocampi of normal SD rats. Images of immunofluorescent staining: GFAP (red), GLT-1 (green), and DAPI (blue). GLT-1 signal in Dex group was more intensive than Saline group. But the phenomenon couldn't be observed after Atipamezole and LY294002 pre-treatment in D and F groups. **(A)** Saline group. **(B)** Dex (1 ug/Kg) group. **(C)** Atipamezole (250 ug/Kg) group. **(D)** Atipamezole (250 ug/Kg) pretreatment + Dex (1 ug/Kg) group. **(E)** LY294002 (300 ug/Kg) group. **(F)** LY294002 (300 ug/Kg) pretreatment + Dex (1 ug/Kg) group. a,b,c,d,e,f 1~4 were 10×, a,b,c,d,e,f 5 was 40×. Scale bars = 50 um.

To intuitive confirm the phenomenon and mechanism *in vivo*, immune fluorescent staining of brian slices were conducted. As we all know, GLT-1 always localized in astrocyte membranes and cytoplasm (26). The results of the double immune fluorescent staining revealed that GLT-1 was co-localized with GFAP in the hippocampus. N = 3 rats in each group. Contrast with the saline treatment group, the positive fluorescent density of GLT-1 was increased after dexmedetomidine treatment at 2 h and there was no difference of fluorescent density between Atipamezole/LY294002 pretreatment group and control group, which was consistent with the results in primary astrocyte cultures (Figure 8).

## Discussion

In this study, using MCAO model, we found that the infarct volume could be reduced by dexmedetomidine and this neuroprotective effects might be related to its upregulation of GLT-1. Furthermore, by using Atipamezole and LY294002, we revealed that dexmedetomidine upregulated GLT-1 expression through PI3K/Akt pathway by activating α2 adrenergic receptors.

Astrocytes play crucial roles in providing energy for neurons, regulating neuronal death and survival after cerebral ischemia (27, 28). It is known that GLT-1 which is crucial for uptaking extracellular glutamate and eventually protecting neurons under pathological conditions (29) happens to be mainly distributed in astrocytes (30). During lots of neurological dysfunction, like epilepsy, Alzheimer's disease, stroke, excitotoxity is a main mechanism of neuron damage which was caused by extracellular glutamate accumulation. Excessive amount of Glu activates many intracellular pathways leading to ATP depletion, loss of membrane potential, dysfunction, apoptosis and necrosis (31). Down-regulation of GLT-1 is reported in various neurological diseases, such as epilepsy, stroke, Alzheimer's disease, and movement disorders (32). GLT-1 knock-down results in increased extracellular glutamate and excitotoxic cell death (33). Based on above studies, GLT-1 was chosen as our primary target.

The MCAO model was used in our study. We measured infarct volume of the rats brains and neurological deficit scores at 2, 6, and 24 h after MCAO, which reflected the neurological damage at hyper-acute phase. In order to get a stable GLT-1 level in brain tissues, we tested GLT-1 expression in brain tissue at 6 and 24 h after the procedure. MRI experiments were used for brain imaging in this study. Acute imaging is crucial in longitudinal pre-clinical stroke studies in order to identify the influence of acute therapies on tissue salvage over time (34).

According to the western blot in our study, GLT-1 expression was significant increased by dexmedetomidine treatment. In addition, GLT-1 expression was also increased about 10% in Sal + MCAO group contrasted with Sal + Sham group, which was consistent with previous research (17). In order to rule out the effects of MCAO *per se* on GLT-1 expression, we used primary cultured astrocytes and rats without MCAO in the later study for potential mechanism of dexmedetomidine. Dexmedetomidine is a highly selective α2 adrenergic receptor agonist (35). To clarify the potential mechanism of GLT-1 upregulation, atipamezole (α2-adrenergic receptor antagonists) (36, 37) was used and we found that the upregulation of GLT-1 was reversed completely by atipamezole, suggesting that dexmedetomidine upregulates GLT-1 by activating α2 adrenergic receptor. PI3K/AKT signaling pathway is crucial in cell proliferation, differentiation, and adaptation (38). Previous studies (16, 39) confirmed the relationship between PI3K/Akt pathway and GLT-1 expression. Phosphorylation of Akt activates the transcription factors—NF-kB and cAMP-response element binding protein (CREB) (40, 41). There are some binding sites of NF-kB and CRE on the GLT-1 promoter, which control the transcription of GLT-1 (42, 43). According to above researches, we started mechanism researches from PI3K/Akt pathway. Our results showed that Akt was activated and phosphorylated to p-Akt by dexmedetomidine. We, then, used LY294002 to inactivate PI3K pathway and found that following the inhibition of p-Akt, GLT-1 upregulation was reversed completely, suggesting PI3K/Akt signaling pathway was involved in dexmedetomidine-mediated GLT-1 increase.

GLT-1 plays a key role in regulation of glutamate transmission. Dysfunction of GLT-1 correlated with various pathologies, such as traumatic brain injury, stroke, Alzheimer's disease. The concept of pursuing GLT-1 to prevent excitotoxicity has made great progress in recent years and some activators like erythropoietin (25), ceftriaxone (31) have been proved. It is now accepted that GLT-1 transporters are a major target to combat neurotoxicity and provide novel potential therapeutic opportunities for the treatment of neurological diseases (44). However, taking GLT-1 as target for therapeutics still remains to be understood, and explored (44).

**Figure 9:**
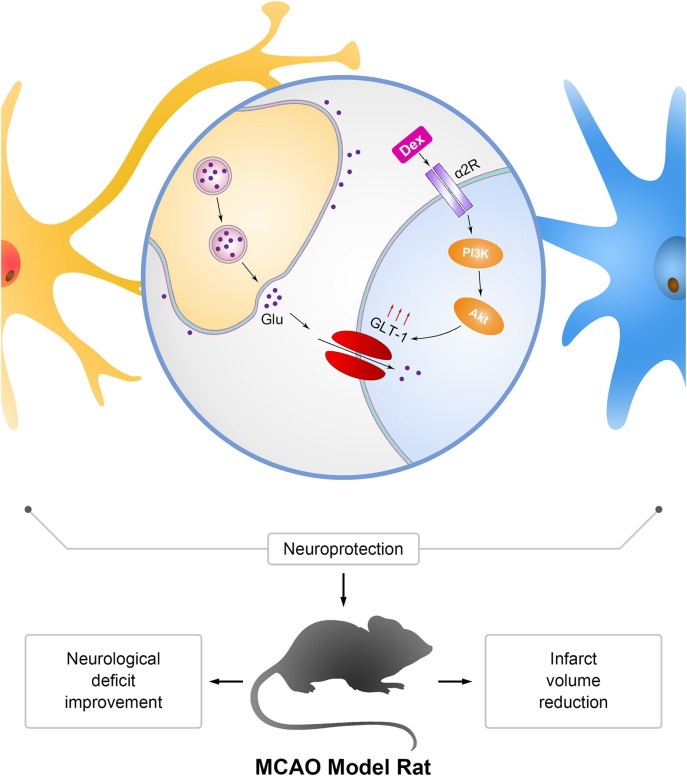
Mechanism of neuroprotection mediated by dexmedetomidine in astrocytes and MCAO rats. Dexmedetomidine (Dex) improved glutamate transporter-1 (GLT-1) expression by activating α2 adrenergic receptor through PI3K/Akt signaling pathway. Upregulation of GLT-1 enhanced the uptaken of extracellular glutamate (Glu) to protect neurons from excitotoxicity. The effect of dexmedetomidine improved the neurological deficit and reduced the infarct volume in MCAO model rats.

Studies in various experimental models revealed that ERK1/2 also involved in the expression of GLT-1 (45) and it remains unclear whether ERK1/2 is involved in dexmedetomidine's neuroprotection mechanism. Further studies should be made in the future. In summary, we demonstrated that the dexmedetomidine improved the neurological deficit and reduced the infarct volume in MCAO model rats. Its neuroprotection on ischemia model might be related to its upregulation of GLT-1. Besides, we revealed that dexmedetomidine enhanced GLT-1 expression via PI3K/Akt pathway by primary astrocytes culture. And dexmedetomidine upregulated GLT-1 mainly by activating α2 adrenergic receptors. We summarized the results in Figure 9. Dexmedetomidine was widely used during perioperative periods in clinical situation. Our results indicated that dexmedetomidine might serve as a new strategy for perioperative neuroprotection, especially in the surgeries with high risk of perioperative cerebral ischemia/reperfusion injury.

## Data Availability Statement

The raw data supporting the conclusions of this manuscript will be made available by the authors, without undue reservation, to any qualified researcher.

## Ethics Statement

This study and protocol was carried out in accordance with the recommendations of Ethics Review Committee for Animal Experimentation of Fudan University and the approve number was 20170223-098.

## Author Contributions

MP and FF designed the experiments. MP and XG carried out the experiments. XL and RS analyzed the experimental results. ZL assisted with the MRI analysis. MP and JC wrote the manuscript.

### Conflict of Interest

The authors declare that the research was conducted in the absence of any commercial or financial relationships that could be construed as a potential conflict of interest.

## Abbreviations

GLT-1: glutamatetransporter-1
Dex: dexmedetomidine
Sal: saline
NDS: neurological deficit scores
MRI: magnetic resonance imaging
MCAO: middle cerebral artery occlusion
CNS: central nervous system
RRID: research resource identifiers
ANDA: abbreviated new drug application
dbcAMP: dibutyryl cyclic adenosine monophosphate
DAPI: 6-diamidino-2-phenylindole
CREB: cAMP-response element binding protein
NF-κB: nuclear factor-kappa B
EGFR: epidermal growth factor receptor
GFAP: glial fibrillary acidic protein
LDH: lactate dehydrogenase
cAMP: cyclic adenosine monophosphate
ERK: extracellular regulated protein kinases
PBS: phosphate buffer saline.
